# The impacts of coping style and perceived social support on the mental health of undergraduate students during the early phases of the COVID-19 pandemic in China: a multicenter survey

**DOI:** 10.1186/s12888-021-03546-y

**Published:** 2021-10-27

**Authors:** Yiman Huang, Xiaoyou Su, Mingyu Si, Weijun Xiao, Hao Wang, Wenjun Wang, Xiaofen Gu, Li Ma, Jing Li, Shaokai Zhang, Zefang Ren, Youlin Qiao

**Affiliations:** 1 School of Population Medicine and Public Health, Chinese Academy of Medical Sciences and Peking Union Medical College, Beijing, China; 2 School of Nursing, Jining Medical University, Jining, Shandong China; 3 Affiliated Tumor Hospital, Xinjiang Medical University, Urumqi, China; 4 Public Health School, Dalian Medical University, Dalian, China; 5 West China School of Public Health, Sichuan University/West China Forth Hospital, Sichuan University, Chengdu, China; 6 Henan Cancer Hospital, Affiliate Cancer Hospital of Zhengzhou University, Zhengzhou, China; 7 School of Public Health, Sun Yat-sen University, Guangzhou, China; 8 Department of Cancer Epidemiology, National Cancer Center/National Clinical Research Center for Cancer/Cancer Hospital, Chinese Academy of Medical Sciences and Peking Union Medical College, 17 South Panjiayuan, Chaoyang District, Beijing, China

**Keywords:** Coping style, Social support, Undergraduate students, Mental health, COVID-19

## Abstract

**Background:**

An increasing number of undergraduate students in China have been reported to have psychological problems. In response to the COVID-19 pandemic, a series of preventive and control measures were implemented, which undoubtedly worsened their psychological health. Coping style and social support were probably important factors that affected the psychological well-being of undergraduate students during the pandemic. This study aimed to explore the effects of coping style and perceived social support on the psychological well-being of college students and relevant risk factors.

**Methods:**

This cross-sectional study was performed in February and March of 2020 by distributing an online questionnaire among undergraduate students from seven geographical regions across China. The questionnaire included sociodemographic information; the 21-item Depression, Anxiety and Stress Scale (DASS-21); the Perceived Social Support Scale (PSSS); and the Simplified Coping Style Questionnaire (SCSQ). For the analyses, t-tests, one-way analysis of variance (ANOVA), the Kruskal–Wallis test and multiple linear regression were utilized. The level of significance was set at *P* < 0.05.

**Results:**

Among 3113 college students, the rates of anxiety, depression and stress symptoms were 13.3, 15.4 and 6.8%, respectively. Increased rates of current smoking and drinking (5.5 and 25.2%, respectively) among undergraduates were identified. The results indicated that the PSSS subscales and SCSQ subscales were significantly associated with DASS-21 scores (*P* < 0.001). Multiple linear regression analysis showed that active coping style and family support were protective factors while passive coping style could aggravate psychological problems among participants (*P* < 0.001).

**Conclusions:**

A remarkable number of college students adopted passive coping strategies to cope with negative feelings, such as smoking and drinking, which were detrimental to their mental health. In contrast, active coping strategies helped improve their psychological well-being. Moreover, family support was particularly important for maintaining their mental health and ameliorating mental health challenges in this major health crisis. Consequently, suitable psychointervention, routine screening for risk behaviors, and provision of further social support are needed for undergraduate students in the COVID-19 pandemic or other emergency public health events.

## Background

On January 30, 2020, the coronavirus disease 2019 (COVID-19) pandemic was declared a public health emergency of international concern (PHEIC) by the World Health Organization, and it has caused severe economic, educational, and social ramifications [1, 2]. During the early epidemic, Chinese health authorities implemented a series of immediate measures to control the disease, and all Chinese people were required to stay at home for isolation; hence, normative teaching activities in universities were forced to stop. These measures, together with fear of infection and uncertainty regarding the consequences of the COVID-19 pandemic, undoubtedly had adverse psychological effects on people, especially university students [3, 4].

Colleges and universities have reported unprecedented numbers of students in psychological distress in recent years. This might be because inexperienced college students are incapable of solving problems in interpersonal relationships, academic challenges, and career development [5]. Several studies indicated that among university students, 9.7% in the eastern and western areas of China, 11.7% in Harbin, 11.8% at 6 universities in Wuhan, 16.8% in Anhui, and 32.82% in the western part of Liaoning experienced depression symptoms [6–10]. The COVID-19 pandemic may increase the stress of undergraduate students or expose them to new stressors that can be frustrating and lead to a range of mental health problems. The frequent contributors to increased stress/anxiety were general uncertainty regarding the pandemic, the precipitous transition to and participation in online classes, and the COVID-19-related influences on their lives [11–13]. The results of a recent study confirmed that students are at high risk of developing depression and suicidality in relation to the COVID-19 outbreak [14]. With the quick closures of universities, students encountered uncertainty and concern about their academic future, social isolation and lack of support, such as limited access to and receipt of mental health services for many vulnerable youths, which were barriers to relieving their stress or negative thinking [15–17].

People’s mental health is affected in many ways. Social support and coping strategies have been proven to be protective factors for mental health in previous studies [18–21]. Social support refers to the provision of practical help, emotional support and information assistance by meaningful groups around an individual when the individual is in distress, such as family members, friends, colleagues, relatives and neighbors [22]. Several studies have shown that younger adults and people with greater social strain or less social support have worse mental health and that perceived social support influences the overall outcome of depression and plays a key role in recovery from affective disorders [23–26]. Coping refers to the thoughts and behaviors people use to manage the internal and external demands of stressful events [27]. Positive coping and negative coping are diametrically opposed coping styles. Individuals who adopted mainly positive coping strategies suffered less emotional distress, while those who adopted negative coping strategies experienced the opposite [28]. Therefore, when people face difficult or complex negative events, the coping style they choose is of the utmost importance, which will affect their psychosocial outcomes and, especially, their mental health.

According to a narrative review, there are numerous pandemic-related mental health risks for children and adolescents, and the subsequent restrictions produce more threats, which may lead to adverse psychological problems [29]. While previous studies have explored the effects of social support and coping style on mental health, they did not identify their impacts on Chinese college students’ mental health during the COVID-19 pandemic, and they had various limitations, such as the use of unstandardized psychological measures, the assessment of single mental health symptoms, and the use of samples that were selected mostly from a single city or university [22, 30–32]. On this basis, the current study is a multicenter (7 cities) study in China, covers a larger population in terms of sample size, and the purpose of it is to better understand the impacts of coping style and perceived social support on mental health and associated risk factors in undergraduate students to inform effective intervention strategies in similar public health events.

## Methods

### Study design and participants

This study is a population-based multicenter cross-sectional online survey that was conducted in seven geographical regions across China. We collected data among college students from various cities by distributing online questionnaires via Wenjuanxing, which is an online crowdsourcing platform in mainland China, in February and March of 2020. The sample size was calculated using a margin of error of 5%, a confidence level of 95%, a response distribution of 50% and a previous estimate rate of depression symptoms of 23.8%, which produced a minimum sample size of 558 [33]. Snowball sampling was used to invite potential study participants. We initially invited investigators from the seven partner institutions, and they forwarded a link to the questionnaire to contacts who they deemed suitable. Finally, data were collected from 4408 participants, and 3113 completed the questionnaire survey (the response rate was 70.62%), which satisfied the minimum sample size.

A brief introduction to the study was displayed to participants, and electronic informed consent was obtained before the start of the questionnaire survey. Upon completion of the informed consent form, they completed the online questionnaire. The inclusion criteria were that the respondents were at least 18 years old and volunteered to complete this online survey. Respondents who were not college students, had a cognitive impairment or felt that using electronic devices was difficult were excluded.

### Measurements

#### Sociodemographics

In the questionnaire, sociodemographic information was collected from all participants, including gender, ethnicity, date of birth, residence place, grade, major, smoking and drinking statuses and health condition.

#### Depression, anxiety and stress scale

Depression, anxiety, and stress symptoms were measured using the Chinese version of the 21-item Depression Anxiety Stress Scale (DASS-21). Respondents rated each item from 0 (does not apply to me at all) to 3 (applies to me very much) on a 4-point rating scale based on their feelings or experiences over the past week. The DASS-21 consists of three subscales (each with 7 items), which measure depression, anxiety, and stress [34, 35]. The DASS-21 has been validated in Chinese populations, and the Cronbach’s alpha values of the three subscales of the DASS-21 were 0.823, 0.811 and 0.861, respectively, and 0.935 overall [35–37]. In this study, the Cronbach’s alpha values of the stress, anxiety and depression subscales were 0.861, 0.812, and 0.826, respectively.

#### Perceived social support scale

The perceived social support scale (PSSS) is a 12-item self-reported inventory that measures the respondents’ perceived support from family, friends and significant others. Each item is rated by participants on a 7-point Likert scale (1 = strongly disagree; 7 = strongly agree), with higher scores indicating higher levels of perceived support. The Chinese version of the PSSS has adequate internal consistency (Cronbach’s α = 0.84) among adolescents, and the Cronbach’s alpha values of the friend, family and significant other subscales were 0.81, 0.92 and 0.83, respectively [38]. In this study, the Cronbach’s alpha values of the total scale and the family, friend, and significant other dimensions were 0.94, 0.88, 0.93 and 0.87, respectively.

#### Simplified coping style questionnaire

Coping style was measured by the Simplified Coping Style Questionnaire (SCSQ). The 20-item Simplified Coping Style Questionnaire measures an individual’s coping style to understand its relationship with psychosomatic health using a 4-point Likert scale (0 = never; 3 = very often) [39]. The instrument has been used frequently in China, with satisfactory reliability and validity [40]. This scale consists of 20 items and is composed of 2 subscales: active coping (12 items) and passive coping (8 items) [41]. In our study, this scale has satisfactory reliability and validity for both active coping styles (Cronbach’s α = 0.89) and passive coping styles (Cronbach’s α = 0.80).

### Statistical analysis

Data were analyzed with SPSS Version 24.0 with the level of significance determined at a 0.05 *p* value. Categorical variables were expressed as frequencies and percent distributions, while continuous variables were presented as means ± standard deviations (SDs). An analysis of descriptive statistics was conducted to examine the demographic and other selected characteristics of the respondents. The t-test, one-way analysis of variance (ANOVA) and Kruskal–Wallis test were used to compare the differences among subgroups. The bivariate Pearson correlation for continuous variables was calculated to determine the association between psychological impact and coping style and social support dimensions. Multiple linear regression was used to explore the psychological impact and its potential factors by adjusting the variables that were significant in a univariate analysis at *P* ≤ 0.10. The level of significance was set at *P* < 0.05.

## Results

### Demographics

Table 1 summarizes the sociodemographic characteristics of the respondents. The study participants were predominantly female (71.4%) and of Han ethnicity (96.8%), and 47.8% were less than 20 years old. Approximately half of the respondents were medical majors, 93.6% were in good health, and 59.5% were rural residents. The attitudes of the participants toward the COVID-19 pandemic included pessimistic (93.8%), neutral (2.0%) and optimistic (4.1%) attitudes. 85.2, 4.2 and 0.4% of the students had confirmed cases in their current city, community or village and among their relatives and friends, respectively. The results showed that 5.5% of the participants were smokers and 25.2% were drinkers. Moreover, of the undergraduates who participated in this study, 13.3% had anxiety symptoms, 478 (15.4%) were depressed, and 6.8% felt stressed.

**Table 1:** The Sociodemographic characteristics of Participants (*N* = 3113)

Variables	N (%)
**Gender**	
Male	889 (28.6)
female	2224 (71.4)
**Age group (Year)**	
18–20	1489 (47.8)
21–23	1444 (46.4)
> 23	180 (5.8)
m ±*SD*	20.83 ± 1.53
**Major**	
Art	1169 (37.6)
Science	589 (28.9)
medical	1355 (43.5)
**Ethnicity**	
Han	3012 (96.8)
Other	101 (3.2)
**Residence place**	
Rural	1851 (59.5)
Urban	1262 (40.5)
**Health condition**	
Fair or poor health	200 (6.4)
Good health	2913 (93.6)
**Isolating status and confirmed cases around**	
Ever been quarantined or isolated	
Yes	1219 (39.2)
No	1894 (60.8)
Have confirmed cases in current city	
Yes	2652 (85.2)
No or not sure	461 (14.8)
Have confirmed cases in current community or village	
Yes	132 (4.2)
No or not sure	2981 (95.8)
Have confirmed cases among relatives and friends	
Yes	12 (0.4)
No or not sure	3101 (99.6)
**Attitudes to COVID-19 epidemic**	
optimistic	2921 (93.8)
neutral	63 (2.0)
pessimistic	129 (4.1)
**Current smoking**	
Yes	171 (5.5)
No	2942 (94.5)
**Current drinking**	
Yes	784 (25.2)
No	2329 (74.8)

### Univariate analysis of anxiety, depression and stress symptoms

According to the univariate analysis (see Table 2), males had higher symptom scores than females, and older students had higher scores than younger students (*P* < 0.05). College students of Han ethnicity had lower scores than minority students. Among them, those who reported being in fair or poor health were more likely to have symptoms of anxiety, depression and stress than those who were healthy (*P* < 0.05). Additionally, students who were required to quarantine or isolate had a higher risk of developing depression symptoms during the lockdown than students without such experience (*P* < 0.05). Respondents who had confirmed cases among relatives and friends were more likely to suffer from anxiety than students whose relatives and friends were not infected (*P* < 0.05). Participants with confirmed cases in their current city had higher depression symptom scores than their counterparts (*P* < 0.05). Students with an optimistic attitude toward COVID-19 were less likely to have psychological problems (*P* < 0.001) than those who were pessimistic or natural. College students who were current smokers or drinkers had higher scores than those who were not (*P* < 0.05).

**Table 2:** Comparison of DASS score among different sociodemographic variables

Variables	N (%)	Anxiety symptoms score		Depression symptoms score		Stress symptoms score	
		($\overline{\boldsymbol{x}}\pm \boldsymbol{SD}$)	***P***	($\overline{\boldsymbol{x}}\pm \boldsymbol{SD}$)	***P***	($\overline{\boldsymbol{x}}\pm \boldsymbol{SD}$)	***P***
**Gender**							
Male	889(28.6)	1.57 ± 2.67	0.039	2.27 ± 3.17	0.032	2.46 ± 3.37	0.009
Female	2224(71.4)	1.36 ± 2.25		2.00 ± 2.80		2.12 ± 2.95	
**Age group (Year)**							
18–20	1489(47.8)	1.31 ± 2.26	0.020	1.91 ± 2.70	0.004	1.96 ± 2.85	< 0.001
21–23	1444(46.4)	1.50 ± 2.46		2.21 ± 3.05		2.44 ± 3.21	
> 23	180(5.8)	1.69 ± 2.62		2.54 ± 3.37		2.62 ± 3.58	
**Major**							
Art	1169(37.6)	1.34 ± 2.20	0.295	1.95 ± 2.67	0.079	2.12 ± 2.90	0.385
Science	589(28.9)	1.43 ± 2.49		2.05 ± 2.89		2.22 ± 3.17	
Medical	1355(43.5)	1.49 ± 2.47		2.21 ± 3.12		2.29 ± 3.18	
**Ethnicity**							
Han	3012(96.8)	1.41 ± 2.37	0.107	2.07 ± 2.91	0.205	2.19 ± 3.05	0.045
Other	101(3.2)	1.84 ± 2.64		2.47 ± 3.07		2.96 ± 3.76	
**Residence place**							
Village	1851(59.5)	1.40 ± 2.34	0.451	2.10 ± 2.95	0.743	2.20 ± 3.06	0.685
City	1262(40.5)	1.46 ± 2.44		2.06 ± 2.87		2.24 ± 3.10	
**Health condition**							
Fair or poor health	200(6.4)	3.27 ± 3.74	< 0.001	4.35 ± 4.76	< 0.001	4.56 ± 4.55	< 0.001
Good health	2913(93.6)	1.30 ± 2.20		1.93 ± 2.68		2.06 ± 2.88	
**Isolating status and confirmed cases around**							
Ever been quarantined or isolated							
Yes	1219(39.2)	1.48 ± 2.35	0.299	2.28 ± 3.06	0.003	2.35 ± 3.13	0.054
No	1894(60.8)	1.39 ± 2.40		1.96 ± 2.81		2.13 ± 3.04	
Have confirmed cases in current city							
Yes	2652(85.2)	1.43 ± 2.36	0.555	2.13 ± 2.98	0.025	2.24 ± 3.10	0.197
No or not sure	461(14.8)	1.36 ± 2.47		1.83 ± 2.54		2.05 ± 2.93	
Have confirmed cases in current community or village							
Yes	132(4.2)	1.86 ± 2.85	0.070	2.40 ± 3.50	0.283	2.63 ± 3.53	0.170
No or not sure	2981(95.8)	1.40 ± 2.35		2.07 ± 2.89		2.20 ± 3.05	
Have confirmed cases among relatives and friends							
Yes	12(0.4)	4.92 ± 4.93	0.031	5.42 ± 5.70	0.067	5.33 ± 5.48	0.074
No or not sure	3101(99.6)	1.41 ± 2.36		2.07 ± 2.89		2.20 ± 3.06	
**Attitudes to COVID-19 epidemic**							
Optimistic	2921(93.8)	1.31 ± 2.18	< 0.001	1.91 ± 2.64	< 0.001	2.85 ± 0.05	< 0.001
Neutral	63(2.0)	3.54 ± 4.77		5.70 ± 6.23		4.83 ± 5.62	
Pessimistic	129(4.1)	3.06 ± 3.67		4.25 ± 4.17		4.67 ± 4.39	
**Current smoking**							
Yes	2942(94.5)	1.92 ±2.90	0.020	2.71 ± 3.78	0.024	2.84 ± 3.61	0.021
No	171(5.5)	1.39 ±2.34		2.05 ± 2.86		2.18 ± 3.04	
**Current drinking**							
Yes	2329(74.8)	1.68 ± 2.55	0.001	2.46 ± 3.13	< 0.001	2.53 ± 3.24	0.002
No	784(25.2)	1.33 ± 2.31		1.96 ± 2.83		2.11 ± 3.01	

### Correlations between anxiety, depression, stress and coping style and perceived social support dimensions

The results of the bivariate Pearson correlation analysis in Table 3 indicated that the PSSS family, friend, and significant other subscale scores and total support score had significant negative associations with the DASS-21 anxiety, depression, and stress symptom scores (*P* < 0.001). The SCSQ active coping dimension also showed significant negative correlations with the DASS-21 anxiety (*r =* − 0.127, *P* < 0.001), depression (*r =* − 0.204, *P* < 0.001) and stress scores (*r =* − 0.146, *P* < 0.001). In contrast, passive coping on the SCSQ had significant positive correlations with the anxiety (*r =* 0.234, *P* < 0.001), depression (*r =* 0.230, *P* < 0.001) and stress symptom scores (*r =* 0.219, *P* < 0.001).

**Table 3:** Correlations between anxiety, depression, stress and coping style and social support dimensions

	DASS Anxiety		DASS Depression		DASS Stress	
	r	***P***	r	***P***	r	***P***
**PSSS family**	−0.211	< 0.001	−0.268	< 0.001	−0.224	< 0.001
**PSSS friends**	−0.160	< 0.001	−0.211	< 0.001	− 0.177	< 0.001
**PSSS significant others**	−0.169	< 0.001	− 0.235	< 0.001	− 0.196	< 0.001
**PSSS total**	− 0.200	< 0.001	− 0.264	< 0.001	− 0.220	< 0.001
**SCSQ AC**	− 0.127	< 0.001	− 0.204	< 0.001	− 0.146	< 0.001
**SCSQ PC**	0.234	< 0.001	0.230	< 0.001	0.219	< 0.001

*DASS* Depression, Anxiety and Stress Scale, *SCSQ* Simplified Coping Style Questionnaire, *PSSS* The Perceived Social Support Scale, *AC* active coping, *PC* passive coping

### Multiple linear regression analysis of factors that influence college students’ mental health

The results of the multiple linear models with the DASS-21 subscale score as the dependent variable are presented in Table 4. Older age was positively related to higher depression (β =0.111, *P* < 0.001) and stress scores (β =0.126, *P* < 0.001). In contrast to fair or poor health, good health was negatively associated with the anxiety, depression and stress subscale scores (*P* < 0.001). Having quarantine or isolation experience was positively correlated with depression (β = 0.298, *P* < 0.05). Having confirmed cases in the current city of residence was a risk factor for depression (β = 0.305, *P* < 0.05). Moreover, the results suggested a positive association between having confirmed cases among relatives and friends and anxiety, depression, and stress symptoms (*P* < 0.001). Participants who had pessimistic or neutral attitudes were more likely to be anxious, depressive and stressed (*P* < 0.001).

**Table 4:** Multiple linear regression analysis of influence factors of anxiety, depression and stress among college students

Variables	Anxiety symptoms score		Depression symptoms score		Stress symptoms score	
	***β*** (95 % ***CI***)	***P***	***β*** (95 % ***CI***)	***P***	***β*** (95 % ***CI***)	***P***
**Age**	0.045 (− 0.006 ~ 0.096)	0.082	0. 111 (0.050 ~ 0.171)	< 0.001	0.126 (0.060 ~ 0.191)	< 0.001
**Gender**	Reference					
Male	0.072 (−0.115 ~ 0.258)	0.452	0.055 (− 0.169 ~ 0.278)	0.631	0.195 (− 0.047 ~ 0.437)	0.114
Female	Reference					
**Health condition**						
Good	−1.417 (−1.740 ~ −1.094)	< 0.001	−1.519 (−1.904 ~ −1.134)	< 0.001	−1.751(−2.168 ~ 1.335)	< 0.001
Fair or not good	Reference					
**Current smoking**						
Yes	−0.051 (− 0.416 ~ 0.314))	0.784	−0.204 (− 0.639 ~ 0.230)	0.357	− 0.113 (− 0.583 ~ 0.357)	0.638
No	Reference					
**Current drinking**						
Yes	0.022 (−0.172 ~ 0.216)	0.824	0.080 (−0.151 ~ 0.311)	0.499	−0.041 (− 0.291 ~ 0.210)	0.750
No	Reference					
**Isolating status and confirmed cases around**						
Ever been quarantined or isolated						
Yes	–		0.298 (0.109 ~ 0.488)	0.002	0.195 (0.010 ~ 0.400)	0.062
No	Reference					
Have confirmed cases in current city						
Yes	–		0.305 (0.046 ~ 0.563)	0.021	–	
No or not sure	Reference					
Have confirmed cases in current community or village						
Yes	–		–		–	
No or not sure	Reference					
Have confirmed cases among relatives and friends						
Yes	3.095(1.850 ~ 4.339)	< 0.001	2.718 (1.236 ~ 4.201)	< 0.001	2.531 (0.926 ~ 4.136)	0.002
No or not sure	Reference					
**Attitudes to COVID-19 epidemic**						
Pessimistic (Yes = 1)	1.284 (0.893 ~ 1.674)	< 0.001	1.681 (1.216 ~ 2.146)	< 0.001	2.016 (1.513 ~ 2.520)	< 0.001
Neutral (Yes = 1)	1.343 (0.784 ~ 1.902)	< 0.001	2.613 (1.947 ~ 3.279)	< 0.001	1.636 (0.915 ~ 2.356)	< 0.001
Optimistic (Yes = 1)	–		–		–	
**Perceived social support**						
Family support	−0.072 (−0.099 ~ −0.045)	< 0.001	−0.097 (−0.129 ~ − 0.065)	< 0.001	− 0.088 (− 0.123 ~ − 0.054)	< 0.001
Friend support	− 0.005 (− 0.039 ~ 0.029)	0.771	0.014 (− 0.027 ~ 0.054)	0.516	0.007 (− 0.037 ~ 0.051)	0.757
Significant others support	0.002 (− 0.033 ~ 0.037)	0.928	− 0.031 (− 0.072 ~ 0.011)	0.149	−0.028 (− 0.073 ~ 0.017)	0.219
**Coping style**						
Active Coping	−0.029 (− 0.046 ~ − 0.013)	< 0.001	−0.068 (− 0.087 ~ − 0.048)	< 0.001	−0.042 (− 0.064 ~ − 0.021)	< 0.001
Passive Coping	0.126 (0.108 ~ 0.144)	< 0.001	0.156 (0.134 ~ 0.178)	< 0.001	0.151 (0.128 ~ 0.175)	< 0.001
**Test statistics**	*R*^2^ = 0.156, AR^2^ = 0.152, *P <* 0.001		*R*^2^ = 0.204, AR^2^ = 0.200, *P <* 0.001		*R*^2^ = 0.162, AR^2^ = 0.158, *P* < 0.001	

Perceived family support and the adoption of an active coping style were protective factors against poor mental health, including anxiety, depression and stress, in college students (*P* < 0.001). Passive coping styles were significantly associated with lower levels of psychological well-being (*P* < 0.001). No significant differences were found in the support from friends and significant others and DASS dimensions (*P* > 0.05).

## Discussion

Experiencing epidemics or natural disasters could lead to long-term psychological disorders in various populations [42, 43]. The rates of probable anxiety, depression and stress symptoms among Chinese undergraduate students in our investigation were 13.3, 15.4 and 6.8%, respectively. Among the symptoms, the prevalence of depression symptoms was higher than the 9.7% prevalence in the eastern and western areas of China, 11.7% prevalence in Harbin, and 11.8% prevalence at 6 universities in Wuhan and lower than the prevalence (32.82%) in the western part of Liaoning, as reported in previous studies during the pre-COVID-19 era [6–8, 10]. Our study was conducted during the early stages of the COVID-19 pandemic and during the traditional “Chinese New Year” festival, when college students stop their work or study plans to enjoy the holiday and might experience less stress and negative emotions than usual. Thus, the rate of depressive symptoms in the current study during this period may be lower than those reported in studies that were conducted in other areas during other periods. In addition, while the smoking rate among all Chinese individuals was approximately 26.6% in 2018 with an overall declining trend in recent years, it is possible that some started to smoke or drink in the extreme situation due to the pandemic as a coping strategy. The current smoking rate of 5.5% (16.3% of male students) and current drinking rate of 25.2% (48.3% of male students) were higher than the rates before the outbreak among college students [44–46].

During the COVID-19 pandemic, tobacco and alcohol misuse are additional public health issues that can cause risk-taking behaviors and mental health issues, especially among undergraduate students, who are still undergoing intellectual and social skill development. Consistent with other studies, students who were current smokers or drinkers were more likely to be anxious, depressive and stressed. In addition, mentally ill people were more likely to be current smokers or substance users than those who did not have a mental illness, and large proportions of smokers (88.7%) and past smokers (97%) reported the onset of smoking before their mental illness was diagnosed in another study [47]. Some of the college students who were current smokers or drinkers might already have had worse psychological health than their counterparts, and the pandemic might have aggravated their problems and caused them to suffer from huge mental health crises. The findings of a recent study revealed that a deterioration of mental health occurred among smokers and binge drinkers during the COVID-19 pandemic and that their tobacco and alcohol consumptions increased since the lockdown [48]. Therefore, the current study highlights the need to screen both mental health status and substance use behaviors among undergraduate students when a similar pandemic or public health emergency occurs. Apart from providing suitable mental health counseling services, family members and school administrators should also pay attention to smoking and drinking behaviors in such circumstances and provide effective smoking cessation and binge drinking interventions if necessary.

Stress is a highly individual reaction, and its effect on mental health varies from person to person. When people face various levels of stress, they might either be motivated and perform effectively or cope inappropriately and be overwhelmed by the stress. The stressors for students while quarantined were diversiform, and the sources of stress included academic pressure, campus relationships, loneliness and COVID-19-related uncertainty [49]. Coping strategy is a critical factor that affects psychological well-being. In this research, passive coping strategies had a negative role in regulating students’ mental health, which were frequently utilized by young people aged 14–35 [50]. During the COVID-19 pandemic, students used their preferred coping strategy to alleviate the increasing amount of stress they experienced, such as seeking help from others (friends, family, school teachers and experts), attempting to solve their problems on their own or coping passively. Negative coping is always emotion-focused or avoidant coping and refers to attempts to avoid actively confronting the problem or to indirectly reduce emotional tension via various behaviors, such as eating or smoking [51, 52]. In the passive coping subscale that was used in the current study, there was an item named “relieve worries by smoking, drinking, taking drugs and overeating”, and 29.4% of the respondents self-reported that they had adopted this strategy. It seems that if students have psychological disorders or are under levels of stress, they are likely to develop health risk behaviors such as the use of cigarettes, liquor, or marijuana, if available. Hence, the higher rates of smoking and drinking among the study participants can be interpreted as indicating that these indirect and evasive behaviors were performed to reduce emotional tension, which may be a sign of negative coping. In contrast, an active coping style positively contributed to adjustment to psychological distress and relief of negative feelings. Coping styles are based on the cognitive evaluation of stressful situations where the relationship between an individual and the stressful situation constantly changes with the individual’s actions [53]. Evidence suggests that depression and anxiety symptoms can, in turn, exert deleterious effects on the ability of late adolescents to effectively cope with daily stressors [54]. It is necessary for interventions to focus on helping students avoid negative coping strategies and cultivate optimistic thinking styles to reduce the risk of the onset of mental health problems.

In line with our hypotheses, family support was a protective factor for the psychological well-being of undergraduate students, while support from friends and others did not significantly affect their well-being, which was inconsistent with previous studies that reported that people could be inspired by friends [30, 55]. As face-to-face interactions and random encounters are minimized due to social distancing measures, it is likely that individuals focus on relationships that are spatially close, most meaningful, or most established [16]. During the early COVID-19 pandemic, almost all undergraduate students were on winter holiday and quarantined or isolated at home. They spent most of their time with their family members and had much less contact with friends or other people. Therefore, we should pay attention to the role of family support in maintaining college students’ mental health in such a special context, particularly among students who live with dysfunctional families or divorced families or orphans who lack sufficient family support, as they may be more prone to develop health risk behaviors and mental illness [56, 57]. Effective communication between parents and their undergraduate children has been proven to play an important role in maintaining good psychological health in terms of self-esteem, socioemotional adjustment and well-being [58]. However, parent-child communication gaps are quite common due to the widespread use of electronic devices, the special life stages of undergraduate students and the advanced society. A lack of communication between parents and their children may be one risk factor that increases their vulnerability if parents use more maladaptive coping strategies or if their family atmosphere is characterized by irritability after the disaster [59]. Parents should have the ability to identify the differences they have with their children and overcome those barriers to understanding each other. Meanwhile, support from health educators and public health specialists should be provided in early intervention programs to promote better mental health and prevent the initiation of health risk behaviors such as substance use.

This survey also explored other risk factors that are related to the psychological problems of undergraduate students. The results indicated that the scores of stress symptoms were significantly higher in minorities than in students of Han ethnicity, which is partly because these poorer minorities are more likely to have more difficulty implementing prevention measures and their overall burdens of disease were heavier [60]. On the other hand, ethnic minorities in remote areas may have more concerns about household income, inconvenience of online teaching activities, scarce living resources and shortages of medical resources. Moreover, areas that are close to the border were in a more severe situation during the pandemic due to cases that were imported from abroad. The results also showed a positive relationship between good physical health and mental health among the participants, and obviously, good physical condition might give participants more power to face emergency public health events and enable them avoid exposure to the dual burden of the COVID-19 pandemic and other chronic diseases [31]. Moreover, it appeared that a worsening of mental health was associated with more worries about one’s family and friends, especially when their friends or family members were infected with COVID-19, which was also consistent with other relevant studies [32, 61].

The current study showed that COVID-19 does have negative psychological effects on college students, and we found that adopting active coping styles can improve their psychological problems. In particular, during the COVID-19 pandemic, family support was a significant protective factor for the psychological well-being of undergraduate students, while support from friends and others was not effective in this case. According to our results, college students should adopt positive coping styles when facing difficulties, setbacks or unexpected events to reduce their probability of suffering from psychological disorders. Social support is one source of positive coping for students, particularly family support in the current study. However, at present, there are an increasing number of dysfunctional families, and many students lack communication with their parents. These students are in urgent need of attention to their psychological status. Therefore, it is crucial for college students to have a positive way of thinking and avoid negative strategies (e.g., tobacco and alcohol use) to cope with challenges, and suitable psychological assistance or intervention is necessary to maintain their well-being and reduce the severity of their psychological problems.

### Limitations

Although this study provided essential findings, it has various limitations. The convenient sampling method and the nature of the survey (internet-based) limit the generalizability of the survey results by generating selection bias. However, during rapidly evolving infectious disease outbreaks, an online survey could produce meaningful results. Second, all the measurements in the current study were assessed by self-report; thus, response bias may exist. Third, females and medical students comprised the majority of the sample in this study, and these two groups may be at additional risk for psychological distress during the COVID-19 pandemic. Therefore, the generalizability of the study results to the general student population may be limited.

## Conclusions

The COVID-19 pandemic resulted in increased psychiatric problems among undergraduate students, and some students used smoking or drinking to cope with difficulties, setbacks or unexpected events during the pandemic, which were negative behaviors that probably aggravated their mental illnesses. Meanwhile, the current study demonstrated that adopting an active coping style and receiving suitable family support can strengthen the ability of college students to regulate their bad emotions, relieve various pressures and cope with psychological disorders. Given the vulnerability of college students who adopt passive coping strategies and have little social support for poor mental health, universities and health authorities should focus on addressing their psychological needs, particularly among students who lack sufficient family support, by screening both mental health status and substance use behaviors, providing high-quality and timely crisis-oriented psychological services and formulating effective strategies to ameliorate their mental health problems, especially during future infectious disease outbreaks.

## Data Availability

The datasets used and/or analysed during the current study are available from the corresponding author on reasonable request.

## Abbreviations

COVID-19: Coronavirus disease 2019
PHEIC: Public Health Emergency of International Concern;
DASS-21: Depression, Anxiety and Stress Scale
PSSS: The perceived social support scale
SCSQ: Simplified Coping Style Questionnaire
AC: Active coping
PC: Passive coping

## References

[1] Mahase E (2020). China coronavirus: WHO declares international emergency as death toll exceeds 200. BMJ (Clinical research ed).

[2] Huckins JF, daSilva AW, Wang W, Hedlund E, Rogers C, Nepal SK (2020). Mental Health and Behavior of College Students During the Early Phases of the COVID-19 Pandemic: Longitudinal Smartphone and Ecological Momentary Assessment Study. J Med Internet Res.

[3] Jiang W, Liu X, Zhang J, Feng Z (2020). Mental health status of Chinese residents during the COVID-19 epidemic. BMC psychiatry.

[4] Bao Y, Sun Y, Meng S, Shi J, Lu L (2020). 2019-nCoV epidemic: address mental health care to empower society. Lancet (London, England).

[5] Zou P, Sun L, Yang W, Zeng Y, Chen Q, Yang H, Zhou N, Zhang G, Liu J, Li Y, Ao L, Cao J (2018). Associations between negative life events and anxiety, depressive, and stress symptoms: a cross-sectional study among Chinese male senior college students. Psychiatry Res.

[6] Chen L, Wang L, Qiu XH, Yang XX, Qiao ZX, Yang YJ, Liang Y (2013). Depression among Chinese university students: prevalence and socio-demographic correlates. PLoS One.

[7] Sun L, Sun LN, Sun YH, Yang LS, Wu HY, Zhang DD, Cao HY, Sun Y (2011). Correlations between psychological symptoms and social relationships among medical undergraduates in Anhui Province of China. Int J Psychiatry Med.

[8] Sun XJ, Niu GF, You ZQ, Zhou ZK, Tang Y (2017). Gender, negative life events and coping on different stages of depression severity: a cross-sectional study among Chinese university students. J Affect Disord.

[9] Hou H, Feng X, Li Y, Meng Z, Guo D, Wang F, Guo Z, Zheng Y, Peng Z, Zhang W, Li D, Ding G, Wang W (2018). Suboptimal health status and psychological symptoms among Chinese college students: a perspective of predictive, preventive and personalised health. EPMA J.

[10] Dong Y, Li H (2020). The relationship between social support and depressive symptoms among the college students of Liaoning, China: a moderated mediated analysis. Psychol Health Med.

[11] Wang C, Pan R, Wan X, Tan Y, Xu L, Ho CS, et al. Immediate Psychological Responses and Associated Factors during the Initial Stage of the 2019 Coronavirus disease (COVID-19) epidemic among the general population in China. Int J Environ Res Public Health. 2020;17(5, 5). 10.3390/ijerph17051729. 10.3390/ijerph17051729 PMC7084952 32155789

[12] Cao W, Fang Z, Hou G, Han M, Xu X, Dong J, Zheng J (2020). The psychological impact of the COVID-19 epidemic on college students in China. Psychiatry Res.

[13] Wang X, Hegde S, Son C, Keller B, Smith A, Sasangohar F (2020). Investigating mental health of US College students during the COVID-19 pandemic: cross-sectional survey study. J Med Internet Res.

[14] Patsali ME, Mousa DV, Papadopoulou EVK, Papadopoulou KKK, Kaparounaki CK, Diakogiannis I (2020). University students’ changes in mental health status and determinants of behavior during the COVID-19 lockdown in Greece. Psychiatry Res.

[15] Prowse R, Sherratt F, Abizaid A, Gabrys RL, Hellemans KGC, Patterson ZR (2021). Coping With the COVID-19 Pandemic: Examining Gender Differences in Stress and Mental Health Among University Students. Front Psychiatry.

[16] Elmer T, Mepham K, Stadtfeld C (2020). Students under lockdown: comparisons of students' social networks and mental health before and during the COVID-19 crisis in Switzerland. PLoS One.

[17] Hertz MF, Barrios LC (2021). Adolescent mental health, COVID-19, and the value of school-community partnerships. Injury Prev.

[18] Yalçın İ (2015). Relationships between well-being and social support: a meta analysis of studies conducted in Turkey. Turk Psikiyatri Derg.

[19] Zhu S, Zhuang Y, Ip P. Impacts on Children and Adolescents’ Lifestyle, Social Support and Their Association with Negative Impacts of the COVID-19 Pandemic. Int J Environ Res Public Health. 2021;18(9). 10.3390/ijerph18094780. 10.3390/ijerph18094780 PMC8124290 33947146

[20] Meshi D, Ellithorpe ME (2021). Problematic social media use and social support received in real-life versus on social media: associations with depression, anxiety and social isolation. Addict Behav.

[21] Fu C, Wang G, Shi X, Cao F (2021). Social support and depressive symptoms among physicians in tertiary hospitals in China: a cross-sectional study. BMC Psychiatry.

[22] Pössel P, Burton SM, Cauley B, Sawyer MG, Spence SH, Sheffield J (2018). Associations between social support from family, friends, and teachers and depressive symptoms in adolescents. J Youth Adolescence.

[23] Zhou Y, MacGeorge EL, Myrick JG. Mental Health and Its Predictors during the Early Months of the COVID-19 Pandemic Experience in the United States. Int J Environ Res Public Health. 2020;17(17). 10.3390/ijerph17176315. 10.3390/ijerph17176315 PMC7503583 32877985

[24] Escobar D, Noll P, Jesus TF, Noll M. Assessing the Mental Health of Brazilian Students Involved in Risky Behaviors. Int J Environ Res Public Health. 2020;17(10). 10.3390/ijerph17103647. 10.3390/ijerph17103647 PMC7277166 32455911

[25] Leskelä U, Rytsälä H, Komulainen E, Melartin T, Sokero P, Lestelä-Mielonen P (2006). The influence of adversity and perceived social support on the outcome of major depressive disorder in subjects with different levels of depressive symptoms. Psychol Med.

[26] Hallgren M, Lundin A, Tee FY, Burström B, Forsell Y (2017). Somebody to lean on: social relationships predict post-treatment depression severity in adults. Psychiatry Res.

[27] Folkman S (2010). Stress, coping, and hope. Psychooncology..

[28] Yan L, Gan Y, Ding X, Wu J, Duan H (2021). The relationship between perceived stress and emotional distress during the COVID-19 outbreak: effects of boredom proneness and coping style. J Anxiety Disord.

[29] Fegert JM, Vitiello B, Plener PL, Clemens V (2020). Challenges and burden of the coronavirus 2019 (COVID-19) pandemic for child and adolescent mental health: a narrative review to highlight clinical and research needs in the acute phase and the long return to normality. Child Adolesc Psychiatry Ment Health.

[30] Humm A, Kaminer D, Hardy A (2018). Social support, violence exposure and mental health among young south African adolescents. J Child Adolesc Ment Health.

[31] Karaca A, Yildirim N, Cangur S, Acikgoz F, Akkus D (2019). Relationship between mental health of nursing students and coping, self-esteem and social support. Nurse Educ Today.

[32] Ma Z, Zhao J, Li Y, Chen D, Wang T, Zhang Z, Chen Z, Yu Q, Jiang J, Fan F, Liu X (2020). Mental health problems and correlates among 746 217 college students during the coronavirus disease 2019 outbreak in China. Epidemiol Psychiatric Sci.

[33] Taherdoost HJP-P (2017). Determining sample size; how to calculate survey sample size.

[34] Lovibond PF, Lovibond SH (1995). The structure of negative emotional states: comparison of the depression anxiety stress scales (DASS) with the Beck depression and anxiety inventories. Behav Res Ther.

[35] Chan RC, Xu T, Huang J, Wang Y, Zhao Q, Shum DH (2012). Extending the utility of the depression anxiety stress scale by examining its psychometric properties in Chinese settings. Psychiatry Res.

[36] Osman A, Wong JL, Bagge CL, Freedenthal S, Gutierrez PM, Lozano G (2012). The depression anxiety stress Scales-21 (DASS-21): further examination of dimensions, scale reliability, and correlates. J Clin Psychol.

[37] Wang K, Shi HS, Geng FL, Zou LQ, Tan SP, Wang Y, Neumann DL, Shum DHK, Chan RCK (2016). Cross-cultural validation of the depression anxiety stress Scale-21 in China. Psychol Assess.

[38] Zimet GD, Powell SS, Farley GK, Werkman S, Berkoff KA (1990). Psychometric characteristics of the multidimensional scale of perceived social support. J Pers Assess.

[39] Folkman SLR (1988). Coping as a mediator of emotion. J Pers Soc Psychol.

[40] Xie Y (1998). Reliability and validity of the simplified coping style questionnaire. Chinese J Clin Psychol.

[41] Kraaij VGN, Maes S (2002). The joint effects of stress, coping, and coping resources on depressive symptoms in the elderly. Anxiety Stress Coping.

[42] Mak IW, Chu CM, Pan PC, Yiu MG, Chan VL (2009). Long-term psychiatric morbidities among SARS survivors. Gen Hosp Psychiatry.

[43] Lee SM, Kang WS, Cho AR, Kim T, Park JK (2018). Psychological impact of the 2015 MERS outbreak on hospital workers and quarantined hemodialysis patients. Compr Psychiatry.

[44] Ye YL, Wang PG, Qu GC, Yuan S, Phongsavan P, He QQ (2016). Associations between multiple health risk behaviors and mental health among Chinese college students. Psychol Health Med.

[45] Sheng Xiong P, Juan Xiong M, Xi Liu Z, Liu Y (2020). Prevalence of smoking among adolescents in China: an updated systematic review and meta-analysis. Public Health.

[46] TAO j-q, ZENG H-w, LIANG L-y (2019). Meta-analysis on smoking status in students from mainland China in recent 5 years. Modern Prev Med.

[47] Asharani PV, Ling Seet VA, Abdin E, Siva Kumar FD, Wang P, Roystonn K, et al. Smoking and Mental Illness: Prevalence, Patterns and Correlates of Smoking and Smoking Cessation among Psychiatric Patients. Int J Environ Res Public Health. 2020;17(15). 10.3390/ijerph17155571. 10.3390/ijerph17155571 PMC7432787 32752263

[48] Guignard R, Andler R, Quatremère G, Pasquereau A, du Roscoät E, Arwidson P, et al. Changes in smoking and alcohol consumption during COVID-19-related lockdown: a cross-sectional study in France. Eur J Pub Health. 2021. 10.1093/eurpub/ckab054. 10.1093/eurpub/ckab054 PMC8083514 33826721

[49] Zhuo L, Wu Q, Le H, Li H, Zheng L, Ma G, et al. COVID-19-Related Intolerance of Uncertainty and Mental Health among Back-To-School Students in Wuhan: The Moderation Effect of Social Support. Int J Environ Res Public Health. 2021;18(3). 10.3390/ijerph18030981. 10.3390/ijerph18030981 PMC7908243 33499409

[50] Liang L, Ren H, Cao R, Hu Y, Qin Z, Li C, Mei S (2020). The effect of COVID-19 on youth mental health. Psychiatric Q.

[51] Billings AG, Moos RH (1981). The role of coping responses and social resources in attenuating the stress of life events. J Behav Med.

[52] Carver CS, Scheier MF, Weintraub JK (1989). Assessing coping strategies: a theoretically based approach. J Pers Soc Psychol.

[53] Lei H, Cheong CM, Li S, Lu M (2018). The relationship between coping style and internet addiction among mainland Chinese students: a meta-analysis. Psychiatry Res.

[54] Wadsworth ME, LEJJoY B, adolescence. Adolescents Coping with Poverty-Related Family Stress: Prospective Predictors of Coping and Psychological Symptoms. 2006;35(1):54–67. 10.1007/s10964-005-9022-5.

[55] Hefner J, Eisenberg D (2009). Social support and mental health among college students. Am J Orthop.

[56] Cheng S, An D, Yao Z, Liu JJ, Ning X, Wong JP, et al. Association between Mental Health Knowledge Level and Depressive Symptoms among Chinese College Students. Int J Environ Res Public Health. 2021;18(4). 10.3390/ijerph18041850. 10.3390/ijerph18041850 PMC7918134 33672872

[57] Caravaca-Sánchez F, Aizpurua E, Stephenson A (2021). Substance use, family functionality, and mental health among college students in Spain. Soc Work Public Health.

[58] Huang CY, Hsieh YP, Shen AC, Wei HS, Feng JY, Hwa HL, et al. Relationships between Parent-Reported Parenting, Child-Perceived Parenting, and Children's Mental Health in Taiwanese Children. Int J Environ Res Public Health. 2019;16(6). 10.3390/ijerph16061049. 10.3390/ijerph16061049 PMC6466341 30909532

[59] Cobham VE, McDermott B, Haslam D, Sanders MR (2016). The role of parents, parenting and the family environment in Children's post-disaster mental health. Curr Psychiatry Rep.

[60] Rothman S, Gunturu S, Korenis P (2020). The mental health impact of the COVID-19 epidemic on immigrants and racial and ethnic minorities. QJM.

[61] Duan L, Shao X, Wang Y, Huang Y, Miao J, Yang X, Zhu G (2020). An investigation of mental health status of children and adolescents in China during the outbreak of COVID-19. J Affect Disord.

